# Supplementary material for: Association between Dietary Acid Load and Chronic Kidney Disease in the Chinese Population: A Comprehensive Analysis of the China Health and Nutrition Survey (2009)
Source: Nutrients. 2024 Jul 29;16(15):2461. doi: 10.3390/nu16152461 (PMC11314566; doi:10.3390/nu16152461)
Supplement: Supplementary file 1 [file nutrients-16-02461-s001.zip › nutrients-3096625-supplementary.pdf]

Supplementary materials

Supplemental Figure S1. Subgroup analysis examined the relationship between DAL and the risk of CKD. (A) Forest plots of the relationship between PRAL and CKD risk; (B) Forest plots of the relationship between NEAD and CKD risk. Abbreviations: PRAL: potential renal acid load; NEAP: net endogenous acid production; Q1 (Percentiles 25); Q2 (Percentiles 50); Q3 (Percentiles 75); Q4 (Percentiles 100).

Supplemental Figure S2. Subgroup analysis examined the relationship between DAL and the risk of CKD. (A) Forest plots of the relationship between PRAL and CKD; (B) Forest plots of the relationship between NEAD and CKD. Abbreviations: PRAL: potential renal acid load; NEAP: net endogenous acid production; Q1 (Percentiles 25); Q2 (Percentiles 50); Q3 (Percentiles 75); Q4 (Percentiles 100).

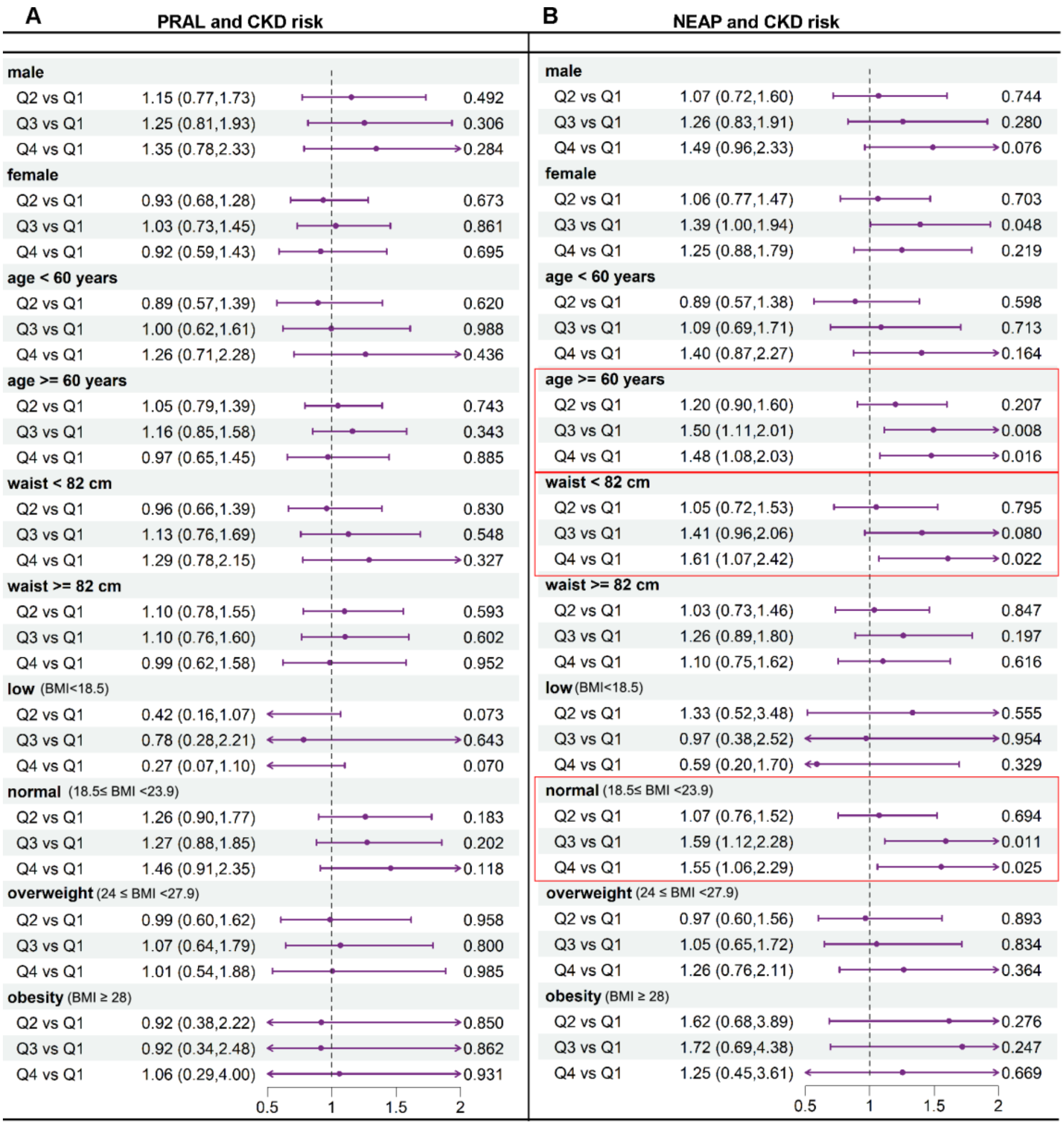

Supplemental Figure S1. Subgroup analysis examined the relationship between DAL and the risk of CKD. (A) Forest plots of the relationship between PRAL and CKD risk; (B) Forest plots of the relationship between NEAD and CKD risk. **Abbreviations:** PRAL: potential renal acid load; NEAP: net endogenous acid production; Q1 (Percentiles 25); Q2 (Percentiles 50); Q3 (Percentiles 75); Q4 (Percentiles 100).

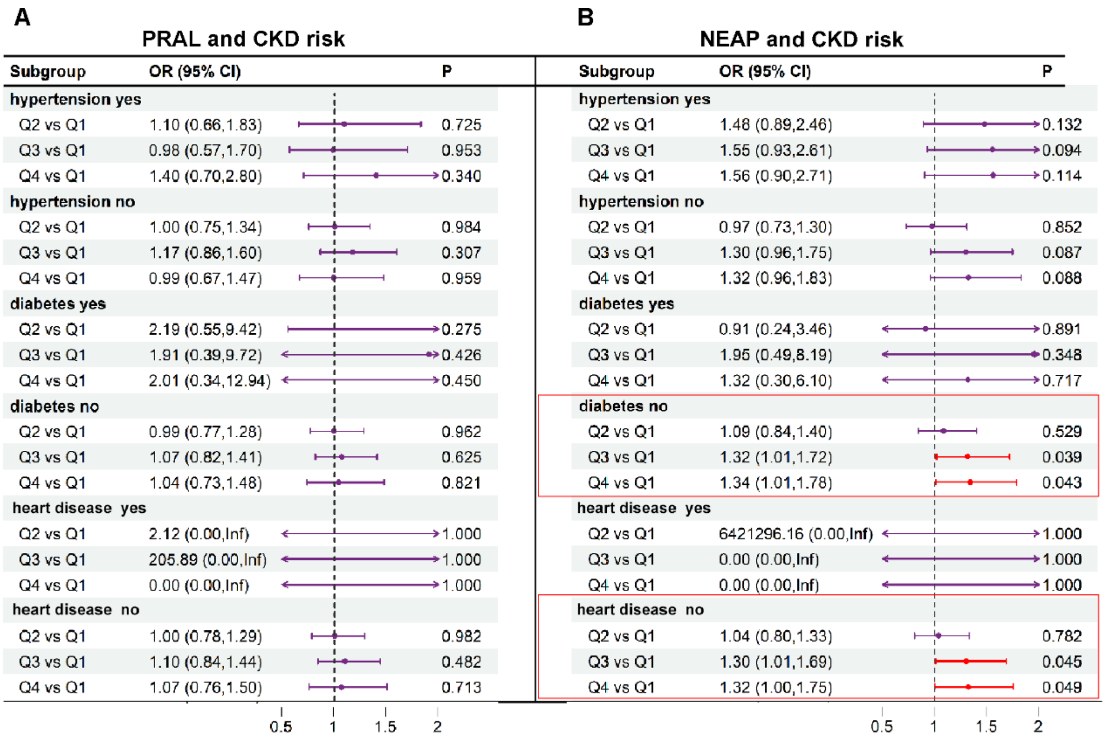

Supplemental Figure S2. Subgroup analysis examined the relationship between DAL and the risk of CKD. (A) Forest plots of the relationship between PRAL and CKD; (B) Forest plots of the relationship between NEAD and CKD. **Abbreviations:** PRAL: potential renal acid load; NEAP: net endogenous acid production; Q1 (Percentiles 25); Q2 (Percentiles 50); Q3 (Percentiles 75); Q4 (Percentiles 100).
